# Complete genome sequence of a highly pathogenic H5N1 avian influenza virus from recent poultry outbreak in Bangladesh

**DOI:** 10.1128/mra.00825-25

**Published:** 2025-11-05

**Authors:** Raduyan Farazi, Md. Touki Tahamid Tusar, Chonda Rani Roy, Roni Mia, Anandha Mozumder, Anupam Das, Jebin Tasmin, S.M. Nazmul Hasan, Md. Shaheen Alam, Sharmin Akter, Sukumar Saha, Tofazzal Islam, Md. Golzar Hossain

**Affiliations:** 1 Department of Microbiology and Hygiene, Bangladesh Agricultural University 54492, Mymensingh, Bangladesh; 2 Virology Laboratory, International Centre for Diarrhoeal Disease Research, Bangladesh 56291, Dhaka, Bangladesh; 3 Department of Physiology, Bangladesh Agricultural University 54492, Mymensingh, Bangladesh; 4 Institute of Biotechnology and Genetic Engineering, Gazipur Agricultural University 198780, Gazipur, Bangladesh; Katholieke Universiteit Leuven, Leuven, Belgium

**Keywords:** highly pathogenic avian influenza virus, H5N1 subtype, complete genome, Bangladesh

## Abstract

Highly pathogenic avian influenza virus (H5N1) continues to cause substantial losses in the poultry industry of Bangladesh, with ongoing genetic evolution. This report presents the complete genome sequence of an H5N1 subtype of avian influenza A virus isolated from a recent outbreak on a commercial layer chicken farm in Bangladesh.

## ANNOUNCEMENT

Avian influenza A viruses (AIVs) are significant pathogens causing respiratory infections in birds and humans (1). They belong to the family *Orthomyxoviridae* and have a segmented, single-stranded, negative-sense RNA genome (2) comprising eight segments that encode 10 or 11 proteins (2). AIVs are classified into 19 hemagglutinin and 11 neuraminidase subtypes (3). In Bangladesh, highly pathogenic avian influenza H5N1 and low-pathogenic avian influenza H9N2 dominate in chickens; novel H5N1 reassortants highlight the need for whole-genome sequencing-based surveillance to track viral evolution (4–6).

In this study, nine chickens from commercial layer farms in Gazipur, Bangladesh, were collected during a suspected AIV outbreak, exhibiting respiratory and gastrointestinal signs. Brain tissues were collected from dead chickens, and 10% tissue homogenates were prepared in phosphate-buffered solution. RNA was extracted using the QIAamp Viral RNA Mini Kit (Qiagen, Hilden, Germany), and cDNA was synthesized using the PrimeScript II 1st Strand cDNA Synthesis Kit (Takara Bio Inc., Shiga, Japan), with random hexamers. Samples were screened using reverse transcription PCR (RT-PCR) with both universal and H5N1-specific primers (7–9). An H5N1 strain was isolated from one representative positive sample by inoculating 10-day-old embryonated chicken eggs via the allantoic cavity and incubating at 37°C for 72 hours (10, 11).

Genomic RNA was extracted from allantoic fluid containing the single virus isolate, and cDNA synthesis and RT-PCR were performed following established protocols (12). The complete genome was sequenced using the Oxford Nanopore MinION platform. Amplicons were purified from the RT-PCR amplified products with AMPure XP magnetic beads (a 0.7× bead-to-sample ratio removed shorter products and residual primers) (12) without further gel- or instrument-based size selection. Libraries were prepared using the Oxford Nanopore Native Barcoding Kit 96 v.14 (SQK-NBD114.96) according to the manufacturer’s instructions, quantified using the Qubit 1× dsDNA Broad Range Assay Kit and Qubit 4 Fluorometer (Invitrogen), and approximately 50 fmol of DNA was loaded onto an R10.4.1 flow cell (FLO-MIN114) using the MinION MK1C (12). Real-time base calling was performed using Guppy v.4.3.4 (13) in MinKNOW (fast base-calling mode), which also handled demultiplexing. Quality filtering used a default *Q* score threshold of 8. Barcode adapters were trimmed using Porechop v.0.2.3 (https://github.com/rrwick/Porechop).

The sequencing run yielded a total of 250,000 raw reads. High-quality reads were assembled into consensus sequences using IRMA v.1.2.0 FLU module (auto-selected, reference-guided approach) which meticulously captured 5′ and 3′ terminal ends (14, 15). The average sequencing coverage across all eight segments of the genome was determined to be greater than 1,000×. The genes were identified and annotated using IRMA’s built-in alignment features, which align assembled contigs to reference sequences (14–16). The pipeline addresses viral variation by iteratively optimizing read gathering and enabling “on-the-fly” reference editing and correction, thereby increasing read depth and breadth without the need for additional reference selection. Default parameters were used for all software unless otherwise noted.

This virus is a reassortant subtype of H5N1 with the PB1 segment of H9N2, consistent with previous reports (17, 18) with genome size 13,076 bp in length, comprising eight segments (Table 1). The complete genome sequence of this H5N1 avian influenza virus from a recent outbreak in Bangladesh will contribute to ongoing surveillance efforts and enhance the understanding of the virus’s genetic evolution, supporting the development of effective control strategies.

**TABLE 1:** Comparison of the nucleotide sequences of the eight gene segments of the isolate A/chicken/Gazipur/Bangladesh/MGH-RF01/2025(H5N1) with its closest relatives in GenBank

Segment	GenBank accession no.	Nucleotide size (bp)	Full genome GC content (%)	Most closely related virus strain (isolate with GenBank accession no.)	Sequence identity (%)
1-PB2	PV961349.1	2,280	44.61	PV739625.1 [A/Quail/Bangladesh/CDIL_AIV_H5N1_54/2025(H5N1)]	98.99
2-PB1	PV961350.1	2,274	42.83	PV739634.1 [A/chicken/Jashore/CDIL_AIV_H5N1_55/2025(H5N1)]	95.51
3-PA	PV961351.1	2,151	43.65	PV739627.1 [A/Quail/Bangladesh/CDIL_AIV_H5N1_54/2025(H5N1)]	99.58
4-HA	PV961352.1	1,704	39.67	PV739628.1 [A/Quail/Bangladesh/CDIL_AIV_H5N1_54/2025(H5N1)]	99.35
5-NP	PV961353.1	1,497	47.49	PV739637.1 [A/chicken/Jashore/CDIL_AIV_H5N1_55/2025(H5N1)]	99.67
6-NA	PV961354.1	1,350	43.70	PV739630.1 [A/Quail/Bangladesh/CDIL_AIV_H5N1_54/2025(H5N1)]	99.41
7-MP	PV961355.1	982	50.10	PV739631.1 [A/Quail/Bangladesh/CDIL_AIV_H5N1_54/2025(H5N1)]	99.59
8-NS	PV961356.1	838	44.63	PV739632.1 [A/Quail/Bangladesh/CDIL_AIV_H5N1_54/2025(H5N1)]	99.64

## Data Availability

The complete genome sequences of all eight segments of the isolate A/chicken/Gazipur/Bangladesh/MGH-RF01/2025 (H5N1) have been deposited in GenBank under accession numbers PV961349.1 to PV961356.1. The raw sequence data are available in the National Center for Biotechnology Information Sequence Read Archive (SRA) under BioProject accession number PRJNA1294791, BioSample SAMN50125628, and SRA PRJNA1294791.
